# Delayed Minocycline Initiation from Symptom Onset and Severe Outcomes in Japanese Spotted Fever: A Single-Center Retrospective Study in Eastern Hiroshima, Japan

**DOI:** 10.31662/jmaj.2025-0593

**Published:** 2026-05-08

**Authors:** Naohiro Oda, Ichiro Takata, Toru Ueki

**Affiliations:** 1 Department of Internal Medicine, Fukuyama City Hospital, Fukuyama, Japan

**Keywords:** Japanese spotted fever, minocycline, treatment delay, older adults, endemic area

## Abstract

**Introduction:**

Japanese spotted fever (JSF) is an endemic tick-borne rickettsiosis in Japan that can cause severe illness and organ failure. Because early tetracycline therapy is crucial for improving outcomes, clarifying the clinical profile of JSF in endemic communities with an aging population and quantifying the impact of treatment delay are clinically important. We aimed to describe the clinical characteristics of patients with polymerase chain reaction (PCR)-confirmed JSF in an endemic region and to evaluate whether delays from symptom onset to minocycline initiation were associated with severe outcomes.

**Methods:**

We conducted a retrospective cohort study in patients with PCR-confirmed JSF admitted to a tertiary hospital in eastern Hiroshima Prefecture between 2011 and 2023. Time intervals were calculated using calendar dates, with same-day events coded as 0 days. The primary outcome was severe JSF, defined as the need for invasive mechanical ventilation and/or renal replacement therapy. Logistic regression was used to assess associations between onset-to-minocycline delay and severe outcomes, adjusting for age and sex.

**Results:**

Forty-two patients (median age, 73.5 years) were included. Severe outcomes occurred in 13 patients (31.0%), and 6 patients (14.3%) died during hospitalization. The median onset-to-minocycline interval was longer in severe cases than in non-severe cases (6.0 vs. 4.0 days; p = 0.035). In multivariable analysis, each 1-day delay in minocycline initiation was associated with higher odds of severe outcomes (adjusted odds ratio, 1.35; 95% confidence interval, 1.01-1.81; p = 0.043).

**Conclusions:**

In this region with endemic JSF and with an older patient population, longer delays from symptom onset to minocycline initiation were associated with severe outcomes. Strategies to shorten onset-to-treatment intervals, including timely clinical examination and empiric tetracycline therapy when JSF is suspected, may improve outcomes in older communities with endemic JSF.

## Introduction

Japanese spotted fever (JSF) is a tick-borne rickettsial infection caused by *Rickettsia japonica*. Since its initial description in 1984 ^(1)^, reported JSF cases have increased steadily, establishing the disease as a significant public health concern in Japan ^(2), (3), (4)^. National surveillance indicates that notification rates remain highest in western Japan and that older adults account for a growing proportion of cases over time ^(2), (3), (4)^. The eastern region of Hiroshima Prefecture, including Fukuyama and surrounding municipalities, is a high-incidence endemic area ^(5), (6)^. In this JSF-endemic zone, many patients with tick bites report recent agricultural work or outdoor activities in mountainous or rural environments, suggesting that infections are frequently acquired during agricultural, forestry, or leisure activities ^(7)^.

The clinical spectrum of JSF ranges from self-limited febrile illness to life-threatening disease characterized by disseminated intravascular coagulation, multiple organ failure, and death ^(8)^. Prior studies have identified older age and organ dysfunction, including thrombocytopenia, hepatic injury, renal impairment, and related laboratory abnormalities, as risk factors for severe JSF ^(9), (10), (11), (12), (13), (14)^. Prompt recognition and early administration of tetracyclines, such as minocycline or doxycycline, are essential for improving prognosis ^(15)^.

Recent retrospective analyses using a nationwide inpatient database have shown that delayed tetracycline initiation after hospital admission is associated with increased mortality in patients with JSF and that adding fluoroquinolones to tetracyclines does not improve outcomes ^(16), (17), (18)^. Hospital-based and regional studies have further indicated that longer intervals from symptom onset to effective antimicrobial therapy correlate with severe disease ^(11), (12)^. In endemic regions with aging populations, both patient delay (symptom onset to first medical visit) and physician delay (first visit to tetracycline initiation) may contribute to adverse outcomes because early manifestations are nonspecific and rash/eschar may be subtle or unnoticed, which can delay recognition at first-contact facilities ^(19), (20)^.

Although delayed tetracycline therapy has been associated with worse outcomes, important uncertainties remain in endemic regions, where patients often initially present to primary care and may experience patient and physician delays. Evidence from region-specific cohorts that quantify the delay from symptom onset to tetracycline initiation and evaluate its association with severe outcomes remains limited. Therefore, we aimed to describe the clinical characteristics of patients with polymerase chain reaction (PCR)-confirmed JSF in an endemic region of eastern Hiroshima Prefecture, Japan, and evaluate whether longer delays from symptom onset to minocycline initiation are associated with severe outcomes. A secondary analysis evaluated the association between age and in-hospital mortality.

## Materials and Methods

### Study design and setting

To evaluate whether delays from symptom onset to minocycline initiation are associated with severe outcomes in JSF, we conducted a retrospective cohort study at Fukuyama City Hospital, a tertiary care facility in eastern Hiroshima Prefecture, Japan. The hospital serves as a referral center for nearby primary care clinics and secondary hospitals in an established JSF-endemic area with higher notification rates than those of many other prefectures in western Japan ^(5), (6)^.

### Patients

We retrospectively reviewed medical records of all patients with JSF who were hospitalized at Fukuyama City Hospital from April 2011 to December 2023 and had a definitive diagnosis confirmed by PCR using blood or eschar/crust specimens. Patients with clinically suspected JSF lacking PCR confirmation and those managed exclusively as outpatients were excluded. Eligibility was not restricted by age. Some patients were initially assessed or treated at referring facilities before transfer.

### Data collection

Demographic information (age, sex, body mass index [BMI]), comorbidities, baseline functional status, and epidemiologic data (exposure history, season) were extracted from electronic medical records. Comorbidities included hypertension, diabetes mellitus, chronic liver disease, cardiovascular disease, chronic kidney disease, chronic respiratory disease, and malignancy, with malignancy categorized as active or in remission. Baseline functional status was classified as independent or dependent in activities of daily living (ADL) on the basis of preadmission documentation.

Clinical data at presentation included level of consciousness assessed using the Japan Coma Scale, admission location (general ward, high care unit [HCU], intensive care unit [ICU]), and the presence of fever, erythematous rash, and eschar. Laboratory results obtained at admission included complete blood count, coagulation parameters, liver and renal function tests, electrolytes, and C-reactive protein (CRP). Treatment-related variables included minocycline and other antimicrobial use (including fluoroquinolones), vasopressors, invasive mechanical ventilation (IMV), and renal replacement therapy (RRT) (continuous renal replacement therapy or intermittent hemodialysis). At our institution, minocycline is routinely used as the standard first-line tetracycline for suspected or confirmed JSF, whereas doxycycline is not routinely used. We also collected data on in-hospital death.

For patients transferred from other hospitals, the dates of symptom onset and initial minocycline administration were primarily obtained from referral letters and transfer summaries, which typically described the clinical timeline and medications initiated at the referring facility. Whenever possible, these dates were cross-checked against admission records.

### Definitions of time intervals

Time intervals were defined using dates in the medical charts. Intervals in days were calculated as differences between calendar dates; events occurring on the same date were coded as 0 days of delay. Because the dataset captured dates but not exact times, the within-day ordering of events (e.g., organ support initiation and minocycline administration) could not always be determined. “Days from symptom onset to first medical visit” was defined as the interval between the onset of JSF-related symptoms (typically fever) and the first presentation to any medical facility. “Days from first medical visit to first minocycline administration” represented the interval from the first medical visit to first minocycline administration. “Days from symptom onset to first minocycline administration” was defined as the interval from symptom onset to the initial dose of minocycline. All patients received minocycline during hospitalization. Time-interval variables were analyzed as continuous measures.

### Severe JSF definition

Severe JSF was defined as requiring IMV and/or RRT at any time during the index hospitalization. This definition was selected because IMV and RRT are objective markers of life-threatening organ failure requiring invasive organ support. Patients who did not receive either therapy were classified as having non-severe disease. Treatment-limitation orders (e.g., do-not-attempt-resuscitation and withholding of life-sustaining treatment) were not systematically abstracted in this retrospective review.

### Outcomes

The primary outcome was severe JSF, as defined above. The secondary outcome was in-hospital death.

### Statistical analysis

Continuous variables were summarized as medians and interquartile ranges (IQRs), and categorical variables as counts and percentages. Group comparisons of the severe and non-severe groups were performed using the Mann-Whitney U test for continuous variables and Fisher’s exact test or the χ^2^ test for categorical variables, as appropriate. Associations between onset-to-treatment time intervals and severe JSF were assessed using univariable logistic regression. A multivariable logistic regression model was constructed with days from symptom onset to first minocycline administration as the primary exposure, adjusting a priori for age and sex. Additional covariates were not included because of the limited number of severe events. A separate univariable logistic regression model evaluated the association between age and in-hospital mortality. Odds ratios (ORs) and 95% confidence intervals (CIs) were calculated, with ORs for continuous time variables expressed per 1-day increase. A two-sided p value <0.05 was considered statistically significant. To assess the impact of potential transfer-related misclassification, we conducted a post hoc sensitivity analysis by repeating the primary multivariable logistic regression after excluding patients transferred from other hospitals. Analyses were performed using R (version 4.3.2; R Foundation for Statistical Computing, Vienna, Austria).

### Ethics approval

The study was approved by the institutional ethics committee of Fukuyama City Hospital (approval number: 809). Owing to the retrospective nature of the study and the use of de-identified data, the requirement for written informed consent was waived. Instead, information about the study was published on the hospital website and posted within the hospital, and patients were provided an opportunity to opt out.

## Results

### Patient characteristics

**Figure 1.**
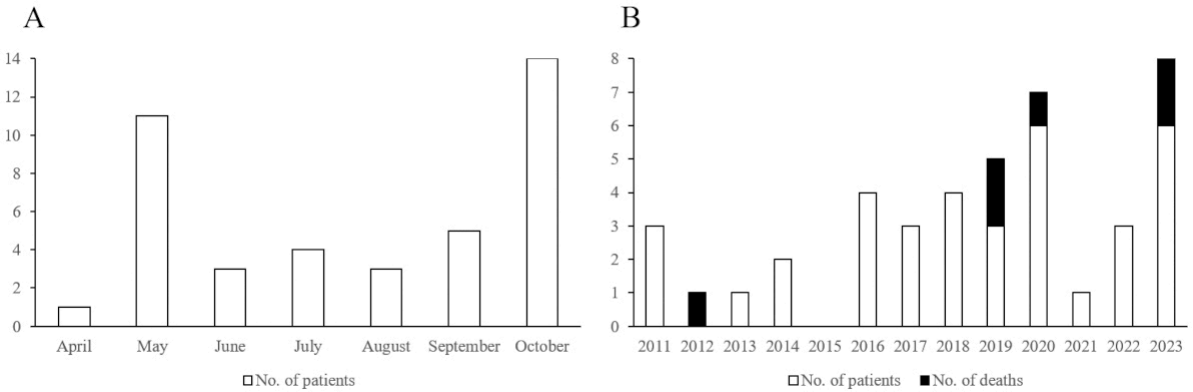
Temporal distribution of polymerase chain reaction-confirmed Japanese spotted fever cases, 2011-2023. (A) Monthly distribution of cases during the study period. (B) Annual number of cases and in-hospital deaths during the study period.

A total of 42 consecutive patients with PCR-confirmed JSF were included. No hospitalized patients with serologically confirmed JSF in the absence of PCR confirmation were identified during the study period. The median age was 73.5 years (IQR, 68.0-80.0 years), and 14 patients (33.3%) were male (Table 1). Figure 1A illustrates the monthly distribution of cases, highlighting the seasonal pattern of JSF in this endemic region. Figure 1B depicts annual case counts alongside the number of in-hospital deaths. Hypertension, diabetes mellitus, chronic liver disease, cardiovascular disease, malignancy (including active disease), chronic kidney disease, and chronic respiratory disease were present in 42.9%, 16.7%, 16.7%, 14.3%, 11.9%, 9.5%, and 4.8% of patients, respectively. None of the patients were receiving maintenance dialysis or had end-stage renal disease before JSF onset. All patients with chronic respiratory disease had asthma, and none had chronic respiratory failure at baseline. Only one patient (2.4%) had an active malignancy. Fever was universal, and erythema and eschar occurred in 95.2% and 66.7% of patients, respectively (Table 1). Most patients were relatively active older adults who regularly entered rural or mountainous areas associated with tick exposure. Nearly all were independent in ADL before onset; only one patient required partial assistance. Regarding care pathways, 39 patients (92.9%) had at least one outpatient visit before arrival at our hospital; 18 (42.9%) had two or more pre-hospital outpatient visits, and 15 (35.7%) were admitted elsewhere and later transferred to our institution.

**Table 1.** Baseline Characteristics on Admission According to Severity.

Variable	All patients (n = 42)	Non-severe (n = 29)	Severe (n = 13)	p-Value
Demographics				
Age, years	73.5 [68.0-80.0]	72.0 [66.0-79.0]	78.0 [70.0-81.0]	0.141
Male sex	14 (33.3%)	11 (37.9%)	3 (23.1%)	0.485
BMI, kg/m^2^	21.8 [21.3-23.7]	21.9 [21.2-23.2]	21.7 [21.4-24.1]	1.000
Comorbidities				
Hypertension	18 (42.9%)	11 (37.9%)	7 (53.8%)	0.501
Diabetes mellitus	7 (16.7%)	5 (17.2%)	2 (15.4%)	1.000
Chronic liver disease	7 (16.7%)	5 (17.2%)	2 (15.4%)	1.000
Cardiovascular disease	6 (14.3%)	3 (10.3%)	3 (23.1%)	0.353
History of malignancy (including active disease)	5 (11.9%)	4 (13.8%)	1 (7.7%)	1.000
Chronic kidney disease	4 (9.5%)	3 (10.3%)	1 (7.7%)	1.000
Chronic respiratory disease	2 (4.8%)	2 (6.9%)	0 (0.0%)	1.000
Health care pathways				
At least one outpatient visit to another institution before presentation	39 (92.9%)	27 (93.1%)	12 (92.3%)	1.000
Two or more pre-hospital outpatient visits	18 (42.9%)	13 (44.8%)	5 (38.5%)	0.748
Admitted to another hospital before transfer	15 (35.7%)	8 (27.6%)	7 (53.8%)	0.163
Clinical presentation				
Fever	42 (100.0%)	29 (100.0%)	13 (100.0%)	1.000
Erythema	40 (95.2%)	28 (96.6%)	12 (92.3%)	0.528
Eschar	28 (66.7%)	21 (72.4%)	7 (53.8%)	0.298

Data are expressed as medians [interquartile range] or numbers (percentages). p-Values were calculated using the Mann-Whitney U test for continuous variables and Fisher’s exact test for categorical variables. BMI: body mass index.

Patients with severe disease tended to be older than those with non-severe disease (median 78.0 vs. 72.0 years), although the difference was not statistically significant (p = 0.141). Distributions of sex, BMI, and comorbidities were similar in the groups (Table 1).

### Laboratory findings on admission

Baseline laboratory results are listed in Table 2. Severe cases had higher white blood cell counts than did non-severe cases. Thrombocytopenia was more pronounced in severe cases (median platelet count 46,000 vs. 99,000/μL, p = 0.002).

**Table 2.** Laboratory Findings on Admission According to Severity.

Variable	All patients (n = 42)	Non-severe (n = 29)	Severe (n = 13)	p-Value
White blood cell count, /μL	7,850.0 [5,397.5-10,667.5]	6,200.0 [4,300.0-9,200.0]	10,700.0 [7,800.0-16,090.0]	0.004
Hemoglobin, g/dL	12.8 [11.4-13.6]	12.8 [11.4-13.6]	12.8 [11.4-13.2]	0.924
Platelet count, /μL	71,000.0 [42,250.0-113,500.0]	99,000.0 [60,000.0-130,000.0]	46,000.0 [23,000.0-59,000.0]	0.002
Prothrombin time activity, %	87.5 [76.2-95.8]	91.0 [81.0-99.0]	77.0 [69.0-89.0]	0.025
Fibrinogen, mg/dL	378.0 [288.8-469.0]	407.0 [321.0-484.0]	342.0 [252.0-411.0]	0.304
FDP, μg/mL	39.0 [21.4-91.2]	29.1 [16.0-66.5]	90.0 [39.0-103.9]	0.028
AST, IU/L	103.5 [69.5-131.8]	84.0 [61.0-119.0]	131.0 [120.0-155.0]	0.006
LDH, IU/L	450.5 [335.2-506.0]	404.0 [304.0-482.0]	525.0 [465.0-696.0]	0.008
Total bilirubin, mg/dL	0.8 [0.5-1.2]	0.6 [0.4-0.9]	1.4 [0.7-2.4]	0.004
ALP, IU/L	219.5 [156.8-297.0]	187.0 [129.0-243.0]	306.0 [287.0-465.0]	<0.001
Albumin, g/dL	2.8 [2.3-3.3]	3.0 [2.5-3.3]	2.3 [2.1-2.5]	0.002
BUN, mg/dL	25.0 [16.8-56.5]	19.8 [14.3-27.4]	71.6 [54.5-78.2]	<0.001
Creatinine, mg/dL	1.2 [0.8-2.1]	0.9 [0.8-1.3]	2.9 [1.7-5.0]	<0.001
CRP, mg/dL	14.3 [9.5-19.2]	11.9 [8.2-15.6]	21.3 [17.9-25.5]	<0.001

Data are expressed as medians [interquartile range]. p-Values were calculated using the Mann-Whitney U test. ALP: alkaline phosphatase; AST: aspartate aminotransferase; BUN: blood urea nitrogen; CRP: C-reactive protein; FDP: fibrin degradation products; LDH: lactate dehydrogenase.

Coagulation and fibrinolytic abnormalities were more pronounced in severe cases, including decreased prothrombin activity and higher fibrin degradation product (FDP) levels, consistent with more advanced coagulopathy (FDP 90.0 [39.0-103.9] vs. 29.1 [16.0-66.5] μg/mL, p = 0.028). Markers of hepatic injury and cholestasis (aspartate aminotransferase, total bilirubin, alkaline phosphatase, lactate dehydrogenase) were higher in severe cases, whereas serum albumin levels were lower, indicating greater hepatic dysfunction and hypoalbuminemia. Renal impairment on admission was markedly worse in severe cases than in non-severe cases (blood urea nitrogen 71.6 [54.5-78.2] vs. 19.8 [14.3-27.4] mg/dL, p < 0.001; creatinine 2.9 [1.7-5.0] vs. 0.89 [0.76-1.27] mg/dL, p < 0.001). CRP levels were also higher in the severe group (21.3 [17.9-25.5] vs. 11.9 [8.2-15.6] mg/dL, p < 0.001), suggesting a greater inflammatory burden (Table 2).

### Time to presentation, treatment, and clinical outcomes

Time intervals, antimicrobial therapy, and outcomes are summarized in Table 3. The median time from symptom onset to the first medical visit was 2 days (IQR, 0-3 days), with no significant difference between severe and non-severe cases (3 [0-4] vs. 2 [0-2] days, p = 0.226). The median time from the first medical visit to the first minocycline administration was 2.5 days (IQR, 1-4 days), with no significant difference between severe and non-severe cases (3 [1-5] vs. 2 [1-4] days, p = 0.610). In contrast, the median time from symptom onset to the first minocycline administration was 5 days (IQR, 3-6 days) and significantly longer in severe than in non-severe cases (6 [5-8] vs. 4 [3-6] days, p = 0.035). All patients received minocycline, and 21 (50.0%) received combination fluoroquinolone therapy, most commonly levofloxacin; the proportion receiving combination therapy did not differ significantly between groups (69.2% vs. 41.4%, p = 0.181) (Table 3).

**Table 3.** Time Intervals, Treatment, and Clinical Outcomes According to Severity.

Variable	All patients (n = 42)	Non-severe (n = 29)	Severe (n = 13)	p-Value
Time intervals				
Symptom onset to first medical visit, days	2.0 [0.0-3.0]	2.0 [0.0-2.0]	3.0 [0.0-4.0]	0.226
First medical visit to first minocycline, days	2.5 [1.0-4.0]	2.0 [1.0-4.0]	3.0 [1.0-5.0]	0.610
Symptom onset to first minocycline, days	5.0 [3.0-6.0]	4.0 [3.0-6.0]	6.0 [5.0-8.0]	0.035
Antimicrobial treatment				
Minocycline use	42 (100.0%)	29 (100.0%)	13 (100.0%)
Combination therapy with fluoroquinolone	21 (50.0%)	12 (41.4%)	9 (69.2%)	0.181
Organ support and outcomes				
ICU/HCU admission	25 (59.5%)	12 (41.4%)	13 (100.0%)	<0.001
Catecholamine use	19 (45.2%)	6 (20.7%)	13 (100.0%)	<0.001
IMV	11 (26.2%)	0 (0.0%)	11 (84.6%)	<0.001
RRT	10 (23.8%)	0 (0.0%)	10 (76.9%)	<0.001
In-hospital death	6 (14.3%)	0 (0.0%)	6 (46.2%)	<0.001

Data are expressed as medians [interquartile range] or numbers (percentages). p-Values were calculated using the Mann-Whitney U test for continuous variables and Fisher’s exact test for categorical variables. The primary severe outcome was defined as the need for IMV and/or RRT during hospitalization. HCU: high care unit; ICU: intensive care unit; IMV: invasive mechanical ventilation; RRT: renal replacement therapy.

Overall, 25 patients (59.5%) required ICU or HCU care; 19 (45.2%) required catecholamines; 11 (26.2%) required IMV; 10 (23.8%) received RRT, and 6 (14.3%) died (Table 3). Thirteen patients (31.0%) met the definition of severe JSF (IMV and/or RRT). All severe cases required ICU/HCU care and catecholamines, and most needed invasive ventilation and/or dialysis. In-hospital deaths occurred exclusively in the severe group (6/13, 46.2%), whereas no deaths were observed among non-severe cases. All patients who died received IMV and/or RRT, suggesting that intensive treatment was not withheld in these cases.

### Association between treatment delay and severe outcomes

In univariable logistic regression, longer delay from symptom onset to the first minocycline administration was associated with higher odds of severe JSF (OR per 1-day delay, 1.32; 95% CI, 1.00-1.73; p = 0.051) (Table 4). In contrast, the delay between the first medical visit and initial minocycline administration was not associated with severe JSF (OR per 1-day delay, 1.06; 95% CI, 0.79-1.41; p = 0.703). After adjustment for age and sex, onset-to-minocycline delay remained associated with severe outcomes (adjusted OR per 1-day delay, 1.35; 95% CI, 1.01-1.81; p = 0.043), whereas age and sex were not independently associated with severe JSF (Table 4). In a post hoc sensitivity analysis excluding transferred patients, the association between onset-to-minocycline delay and severe outcomes remained directionally consistent (adjusted OR per 1-day delay, 1.67; 95% CI, 1.00-2.80; p = 0.051).

**Table 4.** Logistic Regression Analysis for the Primary Severe Outcome.

Predictor	Univariable OR (95% CI)	p-Value	Adjusted OR (95% CI)	p-Value
Symptom onset to first minocycline, per 1-day increase	1.32 (1.00-1.73)	0.051	1.35 (1.01-1.81)	0.043
Age, per 1-year increase	1.05 (0.98-1.13)	0.192	1.05 (0.97-1.13)	0.238
Female sex (vs. male)	2.04 (0.46-9.06)	0.350	2.10 (0.40-11.09)	0.384

The outcome was the primary severe outcome (invasive mechanical ventilation and/or renal replacement therapy). The multivariable model was adjusted for days from symptom onset to first minocycline administration, age, and sex. CI: confidence interval; OR: odds ratio.

### Age and in-hospital mortality

Patients who died during hospitalization tended to be older than survivors. In univariable logistic regression, each 1-year increase in age was associated with higher odds of in-hospital death (OR per 1-year increase, 1.12; 95% CI, 0.98-1.27; p = 0.091). Although not statistically significant, the finding suggests that advanced age may increase vulnerability to fatal outcomes in JSF, even among patients who were largely independent in ADL before disease onset.

## Discussion

In this single-center retrospective study in 42 patients with PCR-confirmed JSF in an endemic area of eastern Hiroshima Prefecture, a longer interval from symptom onset to minocycline initiation was significantly associated with severe outcomes requiring invasive organ support. Each additional day of delay increased the odds of severe JSF by approximately 35% after adjustment for age and sex.

Our results align with and extend prior nationwide and regional studies. Nationwide inpatient database studies evaluated post-admission delay, defined as the time from hospital admission to the initiation of tetracycline. These studies showed that delayed tetracycline treatment after admission is associated with increased mortality in JSF and that adding fluoroquinolones to tetracyclines does not improve outcomes ^(16), (17), (18)^. In contrast, our study focused on the total delay from symptom onset to minocycline initiation, which may better reflect delays along the entire care pathway in primary-care-first endemic settings. Regional studies from Nagasaki Prefecture and Ise Red Cross Hospital similarly reported that longer onset-to-treatment intervals were associated with more severe disease ^(11), (12)^ and suggested that early tetracycline therapy before confirmatory testing may reduce rickettsial mortality ^(12)^. Collectively, our findings complement post-admission evidence by underscoring the clinical relevance of delays from symptom onset to treatment, particularly in endemic regions where many patients have at least one outpatient visit before admission.

The epidemiologic context of this cohort is also notable. Eastern Hiroshima Prefecture, including Fukuyama City, has a high incidence of JSF and an increasing number of cases among older adults ^(5), (6)^. Most patients in this study were older adults who had been independent in ADL before disease onset and were sufficiently active to access mountainous or rural areas where they were at risk of tick exposure. The relatively high frequency of severe outcomes―31.0% requiring invasive organ support and/or causing death―likely reflects the inherent severity of JSF in an aging but active population rather than underlying frailty.

The health care pathways observed in this cohort reflect the regional referral structure. Most patients initially presented to primary care clinics or community hospitals, and nearly all had at least one outpatient visit before being admitted to our hospital. More than one-third were initially hospitalized elsewhere and later transferred. These patterns highlight that in endemic rural areas, timely recognition and referral from first-contact facilities are essential to shorten the overall delay from symptom onset to tetracycline initiation.

Several implications arise along the care pathway. First, the delay from symptom onset to the first medical visit should be minimized. JSF often begins with nonspecific symptoms such as fever, headache, and malaise, and a characteristic rash or eschar may be subtle or missed ^(19), (20)^. Second, clinicians in primary care clinics and secondary hospitals within endemic regions should maintain a high index of suspicion for JSF in patients with fever who present with rash or eschar, particularly in older adults and during periods of increased tick activity ^(15), (19)^. Careful skin examination is important because eschars may be small and easily overlooked ^(15), (19)^. When JSF is clinically suspected, empiric tetracycline therapy should be initiated without waiting for confirmatory PCR or serologic testing ^(12), (15)^. In our cohort, the median interval from the first medical visit to minocycline initiation was 2.5 days and tended to be longer in severe cases, although this difference did not reach statistical significance. Together with nationwide database analyses ^(16), (17), (18)^, these findings support efforts to promote early presentation and prompt initiation of tetracyclines to minimize avoidable delays at each step of care.

Our findings also reinforce the vulnerability of older adults with JSF. Age has consistently been identified as a risk factor for severe illness and death ^(8), (9), (10), (11), (12), (13), (14)^. In our cohort, older age tended to be associated with in-hospital mortality in univariable analysis despite the small number of deaths. All patients who died had received aggressive intensive care, including IMV and in most cases RRT, suggesting that intensive treatment was not withheld in these cases. These observations indicate that in older adults, delayed antimicrobial initiation contributes to irreversible organ injury even with full supportive care. Therefore, from a public health perspective, older residents of JSF-endemic areas should be prioritized for education regarding tick avoidance, early symptom recognition, and prompt consultation.

This study has several limitations. First, time intervals were calculated using calendar dates (same-day events coded as 0 days), and exact within-day ordering of clinical events could not always be determined. This date-based imprecision may have caused nondifferential misclassification, likely biasing associations toward the null. Second, the study was conducted at a single tertiary referral center with a relatively small sample size, thereby limiting precision and generalizability; the proportion of severe cases and mortality may be higher than that of the broader JSF population. Third, symptom onset timing was based on patient self-report, introducing potential recall bias. Fourth, variability in documentation among transferred patients could have affected ascertainment of symptom onset and treatment timing; however, sensitivity analyses excluding transferred patients yielded directionally consistent results, supporting the robustness of the primary finding. Finally, the limited number of severe events reduced statistical power for multivariable analyses, and residual confounding by unmeasured factors cannot be ruled out.

Despite these limitations, this study provides clinically relevant evidence from a well-characterized cohort of patients with PCR-confirmed JSF in an endemic, aging community. By focusing on delays from symptom onset to minocycline initiation, we identified a modifiable factor that spans both patient behavior and health care delivery. Together with nationwide database analyses and regional studies from Nagasaki and Mie ^(11), (12), (16), (17), (18)^, our findings support strategies aimed at shortening onset-to-treatment intervals to prevent severe JSF in older endemic regions.

In conclusion, delays in initiating minocycline after symptom onset were independently associated with severe JSF requiring IMV and/or RRT in an endemic area of eastern Hiroshima Prefecture. Older age was associated with in-hospital death in univariable analysis. These findings underscore the need to improve JSF awareness among residents and clinicians in older endemic communities and to implement strategies that reduce avoidable delays and enable earlier tetracycline therapy.

## Article Information

### Author Contributions (ICMJE)

Naohiro Oda conceived and designed the study, collected the data, performed the statistical analyses, and drafted the manuscript. Ichiro Takata and Toru Ueki critically revised the manuscript for important intellectual content. All authors approved the final version of the manuscript and agree to be accountable for all aspects of the work.

### Conflicts of Interest

None

### IRB Approval

The study was approved by the institutional ethics committee of Fukuyama City Hospital (approval number 809).
